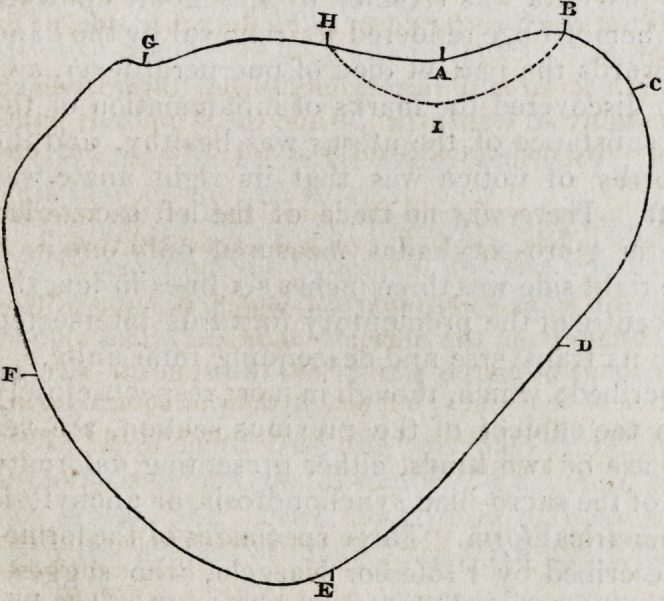# The Obliquely-Contracted Pelvis, with an Appendix Treating of the Principal Defects of the Female Pelvis

**Published:** 1841-01

**Authors:** 


### ART. IX.

*Das Schräg Verengte Becken nebst einem Anhange über die wichtigsten Fehler des weiblichen Beckens überhaupt.* Von Dr. F. C. NAEGELE, &c. &c.—*Mainz*, 1839. 4to, *mit sechzehn Tafeln.*

*The Obliquely-contracted Pelvis, with an Appendix treating of the principal Defects of the Female Pelvis.* By Dr. F. C. NAEGELE, &c. &c.—*Mayence*, 1839. 4to, pp. 124, 16 Plates.

IT is a singular fact that the influence of the pelvis upon labour was one of the last points to which obstetricians directed their attention. We might justly have thought that so frequent and obvious a cause of difficult labour as deformity of the pelvis could not have escaped notice, but before the middle of the past century we find scarcely any mention of the subject.

Fielding Ould in 1742, and Smellie in 1751, were the first to investigate the mechanism of labour, which was soon afterwards still further elucidated by the valuable dissertation of Solayrés de Renhac, "De Partu Viribus Maternis absoluto. Parisiis, 1771." The opinions there expressed were rendered more generally known by Baudelocque, who translated into French the treatise of his late friend and master, and published it in his own work, "De l'Art des Accouchemens." The disputes likewise which arose about the same time with reference to the operation of synchondrotomy led to a juster estimate of the part sustained by the pelvis in natural as well as in difficult labour. Many erroneous notions, however, with regard to the passage of the child through the pelvis still prevailed, and were such as gave rise to serious practical blunders. A mischievously meddling practice, to which Osiander gave the sanction of his high authority, resulted from a belief that many positions of the child's head were unnatural, and that in such cases delivery could not be effected except by manual or instrumental interference. The labours of Professor Naegele have mainly contributed to remove these mistaken ideas. He has too not merely improved and simplified the practice of midwifery in his own country, but his valuable works, and especially his essay on "The Mechanism of Parturition," have obtained for him an European reputation.

The book now before us is worthy of the high reputation of its author. It is, as he informs us, the result of many years of careful observation, and though he has strictly followed the direction *nonum prematur in annum*, he would not even now have published it, but for some circumstances related in the preface. He appears to have ransacked the museums of Europe in search of whatever could elucidate the subject, and we can scarcely suppress a smile at finding a description of the pelvis of an Egyptian mummy adduced in support of his opinions.

The first part of the book, to which we shall entirely confine our notice, treats of "The obliquely-contracted pelvis, that is to say, a pelvis contracted in the direction of one of its oblique diameters with complete anchylosis of the sacro-iliac symphysis of one side, and imperfect formation of the sacrum and os innominatum of that side."

Thirty-four years ago, M. Naegele met with the first two specimens of this malformation of the pelvis. He was struck with their peculiar character, the number of points distinguishing them from pelves deformed by rickets or malacosteon, and with the fact that the persons from whom they were taken had both died during their first labour. In 1813 he found another pelvis closely resembling the two former in every point but one, and in 1825 he saw mention of one similarly malformed in the work of Madame Lachapelle. In 1828 he accidentally discovered another pelvis so like the former ones, that it might have been confounded with them; and he now felt convinced that this peculiar deformity must be the result of certain fixed laws, and could not be a merely accidental production. After a time he succeeded in gaining intelligence of other similar pelves, and was thus enabled on Nov. 24, 1832, at the meeting of the *Gesellschaft fur Naturwissenschaft und Heilkunde*, to describe this as a new and peculiar variety of pelvis. A further account was given of it by the author at the great *Naturforscher Versammlung* at Stuttgart in September, 1834; and in the Heidelb. Klin. Annalen, (Bd. iv. heft 3, s. 449.)*

The peculiar characteristics of this form of pelvis are as follows:

"1. Complete anchylosis of one sacro-iliac symphysis, or perfect continuity of substance of the sacrum with one os innominatum.

"2. Atrophy or imperfect development of one lateral half of the sacrum, and less width of the foramina sacralia anteriora of that side on which the anchylosis is situated.

"3. Less breadth of the os innominatum, and less width of the incisura ischiatica of the same side. That is to say, the distance of the anterior-superior spine of the ilium from the posterior superior spine, as also a line drawn along the brim of the pelvis from the spot where the sacro-iliac symphysis may be supposed to be, along the linea innominata and the pectinea ossis pubis, is shorter than on the corresponding bone of the opposite side. Further, that portion of the hinder part of the inner surface of the os innominatum of the anchylosed side, *superficies auricularis,* by which this bone is connected with the sacrum, is less high or does not reach so far down as on the other side, or as in the well-formed os innominatum.

"4. The sacrum appears driven towards the anchylosed side, towards which also its anterior surface appears more or less turned, while the symphysis pubis is forced towards the opposite side, and consequently is not directly but obliquely opposed to the promontory.

"5. The inner surface of the lateral wall, and the lateral half of the anterior wall of the anchylosed half of the pelvis, are flatter or less excavated than in a normal pelvis.

"6. The other half of the pelvis, namely, that on which the sacro-iliac synchondrosis exists, likewise differs from the normal condition. The side of the pelvis free from anchylosis does not merely share with the opposite side in the abnormal position or direction of the bones; but also in their unnatural form, for a line corresponding to the linea ileo-pectinea will be less curved in its posterior half, more in its anterior half, than natural."

* An abstract of this paper was given by Dr. Rigby in the seventh volume of the London Medical and Surgical Journal. The late Dr. Hamilton, in his Practical Observations, gives a drawing intended to represent a pelvis of this description, which he saw in the collection of Messrs. Pravaz and Guerin, and Dr. Ramsbotham, in his elegant Atlas copies an indifferent representation of a similar pelvis from the work of M. Moreau. Neither he nor Dr. Hamilton, however, seem to have noticed Naegele's paper as reported in the London Journal, nor to be acquainted with Madame Lachapelle's description of a pelvis of this kind in her Pratique des Accouchemens, tom. iii. p. 509.

The consequences which result from what has been already stated, are

"7. *a.* That the pelvis is contracted obliquely, or in a direction crossing a line drawn from the anchylosis to the opposite side, while in the opposite diameter it is not contracted, but in an extreme degree of obliquity is even longer than natural. This may be illustrated by comparing the form of the brim of the pelvis, to an oval figure placed obliquely; the transverse or small diameter of which would correspond to the contracted oblique diameter, and its long diameter to the opposite oblique diameter of the pelvis."

"*b.* That the distance between the promontory and the region above each acetabulum—distantia sacro-cotyloidea—as also the distance from the apex of the sacrum to the spinous process of either ischium, is less on the side where the anchylosis exists.

"*c.* That the distance between the tuber ischii of the anchylosed side and the spina posterior superior of the opposite ilium, as also the distance between the spinous process of the last lumbar vertebra and the anterior superior spine of the ilium of the anchylosed side, is less than the same measurements on the opposite side.

"*d.* That the distance of the under edge of the symphysis pubis, from the spina posterior superior of the ilium of the anchylosed side, is greater than the distance from the symphysis pubis to the same point on the opposite side.

"*e.* That the walls of the pelvic cavity converge somewhat at their lower part, and the arch of the pubes is more or less contracted, consequently approaches the form presented by a male pelvis.

"*f.* That the acetabulum of the flattened side is directed more forward than natural, while that of the opposite side is inclined almost directly outwards." (pp. 7-11.)"

A glance at the accompanying diagram will best explain the nature of the deformity.

The dotted line H I B marks the projection of the promontory.

I, The middle of the promontory.

E, The situation of the symphysis pubis.

G, The situation of the right sacro-iliac symphysis.

C, The point corresponding to where the left sacro-iliac symphysis ought to be.

D F, The situation of the acetabula.

The letters A B C D E F G H mark the circumference of a plane bounded by a line drawn along the upper edge of the symphysis pubis and the linea ilio-pectinea, and continued along the anterior surface of the sacrum.

Professor Naegele observes that all the pelves affected by this deformity bear the most exact resemblance to each other, and that the bones present no mark of rickets, malacosteon, or any other disease, but appear in every respect perfectly healthy. He suggests, as the most appropriate name, the term "*pelvis oblique ovata.*"

In § iii. he proceeds to give a description of all pelves thus deformed, of which he has been able to procure any account: they are thirty-seven in number—two male and thirty-five female; and the history of all (where any history could be obtained) was, with one exception, the same, namely, that the persons were primiparæ, who died undelivered or in consequence of the severity of their labour. The fourteenth case forms the exception; the woman having given birth to six living children with no extraordinary difficulty, and having died at length from the effects of a cold caught four days after her last delivery.

Of all the cases, one only (No. 3) came under the notice of Professor Naegele during the person's lifetime. The particulars of her history are briefly these: A healthy girl, aged nineteen, was admitted into the hospital at Heidelberg at the end of her first pregnancy. She appeared to be well formed, but was thought to halt slightly in her walk. Measurement with Baudelocque's *compas d'épaisseur* gave an external conjugata of seven inches, and the promontory could not be reached even when two fingers were introduced into the vagina. Attention was drawn to the case by the circumstance that the head of the child, instead of being low down, as it usually is in first pregnancies, was situated very high up, and was exceedingly moveable. The liquor amnii escaped two days before any pains were felt, and it was not until the third day after labour had commenced that the head was sufficiently low down to be reached by the forceps. Great difficulty was experienced in extracting the child, which was dead and putrid. The placenta was retained by spasmodic contraction, and the occurrence of hemorrhage rendered its removal by the hand necessary. Five days afterwards the patient died of puerperal fever, and a post-mortem examination discovered the marks of inflammation of the uterine appendages. The substance of the uterus was healthy, and the only point it presented worthy of notice was that its right angle was situated higher than the left. There was no trace of the left sacro-iliac symphysis; the left distantia sacro-cotyloidea measured only one inch ten lines, while that on the right side was three inches six lines in length; and a line drawn from the centre of the promontory forwards intersected the left os pubis, just where its transverse and descending rami unite.

Other pelves are next described; which, though in most respects closely resembling those that form the subject of the previous section, are yet not exactly similar. They are of two kinds, either presenting deformity in shape without anchylosis of the sacro-iliac synchondrosis, or anchylosis of that joint without unsymmetrical form. Three specimens of the former and one of the latter are described by Professor Naegele, who suggests that they probably form the connecting link between the natural pelvis and that species of deformity of which his work treats.

In § vi. the origin of this form of pelvis is discussed; and M. Naegele still inclines to the theory which he adopted at the first, namely, that it is owing to congenital malformation. He assigns the following reasons for this opinion:

"1. The intimate and perfect blending of the sacrum with the ilium, as well in internal structure as in outward appearance; so that, in many instances, the situation of the synchondrosis presents no sign of any previous separation.

"2. The incomplete development of one lateral half of the sacrum in its whole length, as well as the deficient breadth of the os innominatum on the same side, and especially the circumstance that that part where the blending of the sacrum and the os innominatum occurs is shorter longitudinally than the synchondrosis on the opposite side, or in a well-formed pelvis.

"3. Synostoses and deformities are known to occur as original malformations in other bones; and congenital synostosis is usually associated with deformity of the bones, the result of arrest of development.

"4. The striking similarity between the different specimens of this deformity speaks for an identity in their cause.

"5. In no instance can it be proved that any morbid condition of the bones existed, or that any external injury had been inflicted on them, such as might give rise to the deformity." (pp. 65-6.)

Although we cannot admit the force of all the reasons assigned by Professor Naegele, yet we are fully disposed to allow the possibility of this form of pelvis being the result of original malformation. It is well known that instances of want of symmetry in organs naturally symmetrical are by no means unfrequent.* Specimens of oblique skulls are to be found in almost every museum, and we have seen in the anatomical collection at Bonn many specimens of oblique skulls and oblique pelves from the same individual. The existence of obliteration of the sutures and consequent synostosis of the bones of the skull is likewise by no means unusual;† and a case described by Dr. Tourtual, of Münster,‡ and alluded to by Professor Naegele, exhibits a combination of synostosis of all the bones of the cranium with great want of symmetry in its form. The os hyoides is sometimes found unsymmetrical, and one half of the thyroid cartilage is occasionally seen to be larger than the other. Obliquity of the uterus, and even hernia of the ovary, from congenital shortness of one round ligament, sometimes occurs, and the two lateral halves of the uterus may differ much in size, while the orifice of the organ is situated far out of the mesial line of the cervix.§ It is also worthy of note, that in case No. 3, in which there seems to be the best evidence for the shape of the pelvis being congenital, the uterus was higher on the right than on the left side.

Original malformation, however, is not regarded by Professor Naegele merely as being one of several possible causes of obliquity of the pelvis, but he considers that the deformity is in all cases congenital. It is with much hesitation that we venture to dissent from so high an authority, but the evidence which he has adduced in support of his theory does not appear to us conclusive.

We apprehend that few persons would feel disposed to regard an anchylosis as congenital merely on account of the intimate blending of two bones, and partial absorption of bone is a very frequent attendant upon the process of anchylosis. An excellent illustration of this is pre-

* Mehlis, De Morbis Hominis dextri et sinistri; 4to, Gottingæ, 1817. Delpech, De l'Orthomorphie; 8vo, Paris, 1828, tom. i. art. v. And Weber, in V.-Gräfe und V. Walther's Journal, Bd. 4, Heft 4, s. 694.

† Van Döveren, Observat. Osteolog. in his Specimen Observat. Acad.; 4to, 1765, p. 194. Sandifort, Observat. Path., lib. i. cap. vii., and lib. ii. cap. vi.

‡ Med. Zeitung der Verein für Heilkunde, 1836, s. 175.

§ Deneux, Récherches sur la Hernie de l'Ovaire; 8vo, Paris, 1813. Fr. Tiedemann, Von der schiefen Bildung und schiefen Lage der Gebärmutter; 4to, 1840, Taf. ii. iii. iv.

sented by a preparation of anchylosis of the head of the femur into the acetabulum in the museum of University College; and similar specimens may be found in almost every anatomical collection. In the museum of Guy's Hospital are several preparations of anchylosis of the sacrum with the os innominatum, in some of which scarcely a trace remains of the existence of the symphysis. It should also be borne in mind, that perhaps no joint in the body is so liable to become anchylosed as the sacro-iliac synchondrosis.*

His second argument is much more weighty; but still, since it is a notorious fact that a bone may become atrophied from a variety of causes in the adult,† we think that other proofs than the mere assertion that it is always congenital in this case, ought to be brought forward by Professor Naegele.

The work of Madame Lachapelle‡ affords instances of great contraction of one half of the pelvis from causes operating long after birth. In a case of unreduced luxation of the femur, in which the bone formed for itself a new acetabulum, contraction of the cotyloid cavity was found to have taken place, with shortening of the ischium, and contraction of the corresponding half of the brim of the pelvis. In another instance amputation of the left thigh was followed by shrinking of the right side of the brim of the pelvis to half its natural size, while the whole pelvis was rendered oblique, and the pubis was forced towards the left side. A similar case is mentioned by Herbiniaux.

The last reason assigned by the author, namely, that no morbid condition of the bones existed, and that no injury had been inflicted, appears conclusive until we examine the grounds upon which he rests his statement. It will then be seen, that of thirty-one of the thirty-seven cases described, the history is altogether unknown. Of the remaining six, in all of which it is said that the persons were well formed, one only came under M. Naegele's own observation during the lifetime of the individual. Of these six, two were noticed to have been unusually backward in learning to walk; and two, including one of those just mentioned, presented curvature of the lumbar vertebræ. In two, the state of the lumbar vertebræ is not mentioned, and in two only they were stated to have been found perfectly straight on examination of the pelvis. In twenty-five of the remaining thirty-one, of which no history is known, no part of the spine appears to have been preserved with the pelvis. In four, there was a lateral curvature of the lumbar vertebræ, in one instance they were straight, and in one it is not stated whether they were straight or crooked. Three of the pelves presented marks of hip-joint disease, or of ulceration about the acetabulum.

* Créve, Von den Krankheiten des weiblichen Beckens, 4to, Berlin, 1795, s. 165. In his own museum, and in those of the Wenzels, of Weidmann, and of Sömmering, Créve met with 70 specimens of anchylosis of both ossa innominata with the sacrum, and 250 of anchylosis on one side. Of these latter 101 were from the male, 149 from the female subject: 171 were anchylosed on the right side, 70 on the left. He likewise states that in other collections he saw quite as many specimens as those above enumerated, and feels himself therefore warranted in stating "that this joint is more liable to anchylosis than any other in the pelvis, or indeed in the whole body."

† J. Shaw, on Distortions of the Spine and Chest; London, 1823, pp. 6-8.

‡ Traité des Accouchemens; Paris, 1825; tom. iii. p. 413.

The assertion, then, that no morbid condition of the bones existed can be understood as referring only to the absence of rachitis or malacosteon. But we are left almost completely in the dark as to the state of the spinal column, except in the few instances where the plates assist us by representing two or three lumbar vertebræ in connexion with the pelvis. It is, however, of great importance to ascertain the condition of the spine, since in a great number of cases of common lateral curvature the pelvis is contracted obliquely and presents almost exactly the appearance of the *pelvis oblique ovata*, except that the sacro-iliac synchondrosis is not anchylosed.* It will be remembered, too, that curvature of the lumbar vertebræ existed in most instances where their condition was at all noticed. In three of the cases there were the marks of previous hip-joint disease; and Dr. Betschler, who thinks, as we do, that Dr. Naegele's arguments are not conclusive, mentions in his review of this work in Busch's Journal, a case where disease of the bones deformed the pelvis exactly in the way which Professor Naegele regards as congenital. This is not the place to enter upon a lengthened discussion of the subject; but we hope that in all future cases of oblique pelvis, the condition of the spinal column will be more carefully examined than it has been hitherto. At present it appears to us that proof has not been brought forward sufficient to show that this deformity is in all cases congenital, and that it is never produced by causes acting after birth.

In the following section the author examines the influence of this form of pelvis upon labour. It operates in two ways: first, by directly retarding the passage of the head, and next, by impeding those rotations which the head makes in its passage through a well-formed pelvis. Of course, the original size of the pelvis will greatly modify the degree of difficulty thus produced. The practitioner should also recollect that it is not the contracted brim of the pelvis which is the sole impediment to the passage of the head, but that the convergence of its walls from above downwards, increases the opposition to the head in proportion as it descends. If, in a case of this sort, it were thought necessary to apply the forceps, the peculiar form of the pelvis should be borne in mind; or, if the child were turned, attention should be paid in extracting the head to bring its long diameter into correspondence with the longest oblique diameter of the pelvis.

In § viii. Professor Naegele makes some very just observations upon the difficulty of discovering this form of pelvis in the living subject, and on the insufficiency of the ordinary modes of pelvimetry for the purpose. In all those cases the history of which is known, the existence of the deformity was not suspected before labour began. Not only is the measurement with Baudelocque's *compas d'epaisseur* quite unsuited to detect the malformation, but even vaginal exploration, which Madame Lachapelle regarded as a never-failing test of a natural pelvis, would totally mislead. Inability to reach the sacrum with the finger

* Proof of this is afforded by the measurements appended to Choulant's Decas Pelvium Spinarumque Deformatarum; Lipsiæ, 1818. Decas Secunda; 1820, 4to. Jörg also gives a good illustration of this fact in his work, Ueber die Verkrümnungen des menschlichen Körpers; Leipsig, 1840, 4to, Pl. i, fig. 1. The same may be observed on a careful examination of anatomical collections; and a spine and pelvis in the museum of Guy's hospital, afford a striking confirmation of our statement.

instead of affording a proof that the pelvis was well formed, would, in this instance, rather indicate a high degree of deformity.

The old methods of pelvimetry having been seen to be useless, M. Naegele made many experimental measurements, and now proposes to ascertain,

"1. The distance from the tuber ischii of the one side to the spina posterior superior ossis ilii of the opposite side.

"2. From the spina anterior superior of one ilium, to the spina posterior superior of the opposite side.

"3. From the processus spinosus of the last lumbar vertebra, to the spina anterior superior of each ilium.

"4. From the trochanter major of one side to the spina posterior superior ossis ilii of the opposite side.

"5. From the middle of the under edge of the symphysis pubis to the spina posterior superior of each ilium." (p. 74.)

The author observes that all the above-named points are easy of access, and may be readily recognized, and that the employment of all complicated instruments to ascertain the distance between them is quite unnecessary, since a common *compas d'epaisseur* will answer every purpose. The chief recommendation of all is, that these dimensions are nearly or exactly the same on both sides in symmetrical pelves, but differ in the oblique pelvis quite sufficiently to enable us to ascertain the existence of deformity. In proof of this he gives tables of the measurements of eight oblique pelves in his own museum.

Our remarks have extended to such a length that we must defer to another time any examination of the appendix which occupies the last thirty pages of the book. The reader will find there much valuable information upon deformities of the pelvis, which he would search for in vain elsewhere.

The plates which illustrate the work are some of the most beautiful specimens of the lithographic art we have ever seen.

---

## Figures and Tables

**Figure f1:**